# Ten Vestibular Tools for Primary Care

**DOI:** 10.3389/fneur.2021.642137

**Published:** 2021-02-12

**Authors:** Otto R. Maarsingh, Vincent A. van Vugt

**Affiliations:** Department of General Practice, Amsterdam University Medical Center (UMC), Vrije Universiteit Amsterdam, Amsterdam Public Health, Amsterdam, Netherlands

**Keywords:** vestibular symptoms, vertigo, dizziness, primary care, general practice, diagnosis, treatment, prognosis

## Introduction

**Figure 1:**
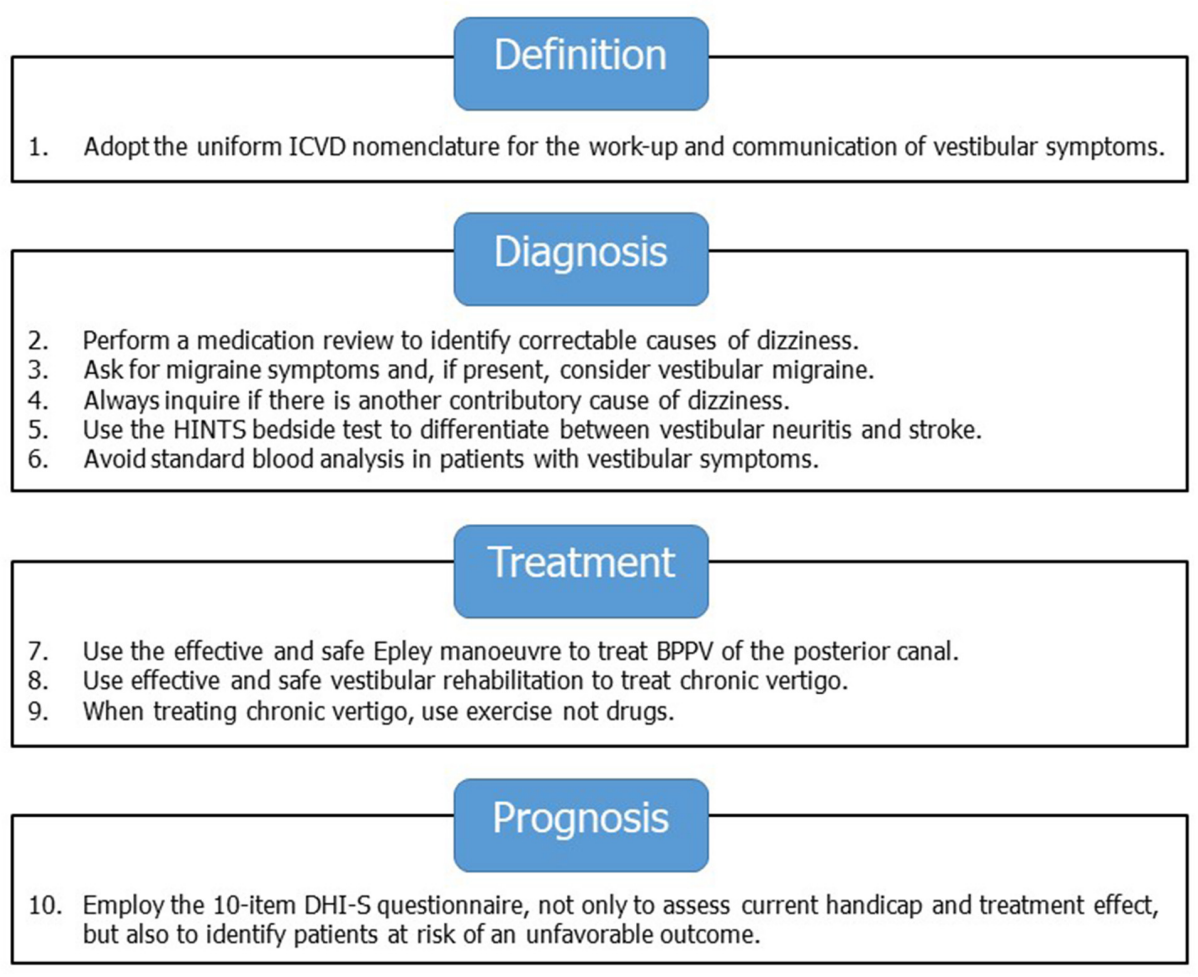
Ten vestibular tools for primary care.

Although primary care physicians (PCPs) regularly encounter patients with dizziness or vestibular symptoms, they often consider these patients as difficult, challenging or even heartsink (1, 2). Given the current scientific evidence and available “vestibular tools,” this is unnecessary. We will provide ten vestibular tools that should not be missed, following definition, diagnosis, treatment, and prognosis, respectively (Figure 1).

## Definition

When approaching a potentially complex problem, the use of a uniform nomenclature is crucial. To date, most primary care guidelines use the typology of Drachman and Hart (3). This typology distinguishes four dizziness subtypes, i.e., vertigo (rotational dizziness), presyncope (lightheadedness), disequilibrium (unsteadiness when walking), and non-specific dizziness. The Drachman-Hart typology is primarily based on how patients describe the nature of their symptoms, assuming that this will provide etiological insight, and therefore, diagnostic guidance (4, 5). However, both doctors and patients use the term “vertigo” differently (6–8), patients are inconsistent when describing their symptoms (7), the identified subtype does not reliably match the suggested etiology (5, 9), and regularly patients have more than one dizziness subtype (10). Therefore, it is time to leave the Drachman-Hart typology and to adopt a more accurate and uniform way to describe vestibular symptoms. The Bárány society, the leading international organization for clinicians and researchers involved in vestibular medicine, previously realized such a nomenclature: the International Classification of Vestibular Disorders (ICVD) (11, 12). The ICVD identifies four main vestibular symptoms, i.e., dizziness (“the sensation of disturbed or impaired spatial orientation without a false or distorted sense of motion”); vertigo (“the sensation of self-motion when no motion is present or the sensation of distorted self-motion during normal head movement”); vestibulovisual symptoms (“visual symptoms that result from vestibular pathology or visual-vestibular interaction”); postural symptoms (“balance symptoms related to maintenance of postural stability, occurring only while upright—seated, standing, or walking”) (13). These vestibular symptoms are not specific in terms of etiology, not overlapping, and not hierarchical (a single patient can experience multiple symptoms) (13). When assessing a patient with vestibular symptoms, the Bárány society recommends to focus on timing (onset, duration, and evolution of symptom) and triggers (actions, movements, or situations that provoke onset of symptoms) (11, 12). Combining the mentioned vestibular symptoms with timing and triggers results in three vestibular syndromes, i.e., acute vestibular syndrome (AVS), episodic vestibular syndrome (EVS), and chronic vestibular syndrome (CVS). AVS is defined as acute-onset, continuous vertigo/dizziness, lasting days to weeks, generally including symptoms that suggest new dysfunction of the vestibular system (like vomiting, nystagmus, and severe postural instability). Disorders presenting with AVS include vestibular neuritis, labyrinthitis, stroke affecting vestibular structures, and traumatic vestibulopathy. EVS is defined as transient vertigo/dizziness lasting seconds to hours, generally including symptoms that suggest temporary dysfunction of the vestibular system (like nausea, nystagmus, and sudden falls). Disorders presenting with EVS include vestibular migraine, benign paroxysmal positional vertigo, Menière's disease, and panic attacks. CVS is defined as chronic vertigo/dizziness lasting months to years, generally including symptoms that suggest persistent dysfunction of the vestibular system (like oscillopsia, nystagmus, and gait unsteadiness). Disorders presenting with CVS include poorly compensated vestibulopathy, bilateral vestibulopathy, and persistent postural perceptual dizziness (13). In short, the ICVD nomenclature provides an essential tool for the work-up and communication of vestibular symptoms in primary care (tool #1).

## Diagnosis

Up to 40% of patients presenting with vestibular symptoms in primary care remain undiagnosed (14, 15). Although this is not unusual for comparable reasons for encounter (like tiredness), we firmly believe it is possible and necessary to reduce the number of undiagnosed dizzy patients in primary care. An accurate diagnosis starts with thorough history taking, focusing on symptom characteristics, timing, and triggers according to the ICVD.

During history taking, the importance of a medication review is apparent. Although an adverse drug effect is a rare cause of vertigo/dizziness in younger patients, it is much more prevalent and regularly missed in older patients. Previous research studies showed that Dutch PCPs scarcely reported adverse drug effect as a cause of dizziness in older patients (1–3%) (14, 15), whereas a diagnostic panel study among the same population found a much higher proportion (25%) (10). Drug-induced vertigo can be caused by aminoglycosides, azithromycin, pregabalin, mefloquine, and α-blockers, whereas drug-induced dizziness can be caused by anticonvulsants, antidepressants, anti-psychotics, β-blockers, Calcium channel blockers, antiarrhythmics, diuretics, vasodilators, anxiolytics, and antispasmodics (16–18). As a medication review costs little time and may provide much insight (i.e., clues for intervention), it should not be missed in the diagnostic phase (*tool #2*). During such a review, a practical guide may help to rapidly identify potential adverse drug effects (19).

A more common cause of episodic vertigo is vestibular migraine (VM). This is a migraine variant with vestibular symptoms and poorly understood pathophysiology. Despite its prevalence and high impact on healthcare cost and utilization, VM remains clinically underdiagnosed (20). The diagnostic criteria for VM include the presence or history of migraine, at least five episodes with vestibular symptoms of moderate or severe intensity and at least 50% of episodes associated with migraine features (i.e., headache, motion sensitivity, photo- or phonophobia, or visual aura) (21). We recommend physicians to structurally ask for migraine symptoms (*tool #3)*, and, if present, consider vestibular migraine (VM).

Another small but effective tool, especially regarding older patients, is to incorporate the following question in your diagnostic work-up: Is there another contributory cause of dizziness? (*tool #4*) According to a diagnostic panel study among 417 older dizzy patients in Dutch primary care, 62% had two or more contributory causes of dizziness (10). Among a consecutive cohort of 621 patients in tertiary care (average age 56 years, range 11–90 years), 30% of dizzy patients had more than one diagnosis (22).

If the history reveals red flags (e.g., neurological symptoms, new headache, or acute deafness) (23), it is important to minimize the probability of a central cause of dizziness. However, when sharpening one's diagnostic tools, population awareness is crucial: the prior probability of a central cause of dizziness in a primary care population is very low compared to secondary/tertiary care. In a study cohort that consisted of patients hospitalized with isolated vertigo, the risk for stroke during 4-year follow-up was 3.01-times higher compared to the general population; vertigo patients with three or more risk factors (including age >55 years, male gender, hypertension, diabetes, coronary artery disease, and hyperlipidemia) even had a 5.51-fold higher for stroke (24, 25). However, in a surveillance study among patient presenting with dizziness symptoms to the emergency department, only 0.7% with isolated dizziness symptoms had a stroke/TIA (26). According to the ecology of medical care (27), this proportion will be even lower for patients presenting with the same symptoms in primary care. In case of an acute vestibular syndrome (i.e., rapid onset of vertigo, nausea/vomiting, and gait unsteadiness in association with head-motion intolerance and nystagmus) another promising tool comes in: the three-step Head Impulse–Nystagmus–Test of Skew (HINTS) exam (HINTS; *tool #5*). The HINTS exam is a simple bedside test that is relatively easy to learn (https://medicinetoday.com.au/vertigovideos). The HINTS exam can help differentiate between vestibular neuritis and stroke, because the presence of any of three oculomotor signs (normal horizontal head impulse; gaze-direction nystagmus; or skew deviation) indicates a central cause of acute vestibular syndrome. A recent systematic review revealed that the HINTS exam has a pooled sensitivity of 96% and specificity of 71% to detect stroke (28), which indicates an even higher diagnostic accuracy than early MRI (29).

When revising one's diagnostic tools, it is important to reconsider overrated tools. According to an observational study (*n* = 2,812), Dutch PCPs performed blood analyses in 22% of older patients presenting with dizziness (15). Until present, there is no scientific evidence that standardized blood analysis has additional value during the work-up of vestibular symptoms. Among 4,538 patients included in etiologic studies, laboratory abnormalities that explained dizziness were limited to three patients with electrolyte disturbances, 11 with glucose disorders, 11 with anemia, and one with hypothyroidism (30). In a community based study, the results of standardized blood analysis among 149 dizzy subjects and 97 controls did not differ (31). Therefore, use blood tests only on a strict medical indication and avoid standard blood analysis in patients with vestibular symptoms (*tool #6*).

## Treatment

A very rewarding vestibular tool is the Epley maneuver (*tool #7*). This is a relatively simple, safe, and highly effective treatment for the most prevalent cause of episodic vertigo, i.e., benign paroxysmal positional vertigo of the posterior canal—a diagnosis that can be confirmed by using the Dix-Hallpike test (https://www.youtube.com/watch?v=kEM9p4EX1jk&feature=youtu.be) (32). Despite its proven effectiveness, PCPs have not yet embraced the Epley maneuver. During a survey among 426 PCPs, only 57% used the Epley maneuver. The most common reason (50%) for PCPs not to use the maneuver was that they did not know how to perform the technique (33). The second reason (30%) was not being convinced of its effectiveness. Both deserve reconsideration, as the Epley maneuver can be easily learned (https://medicinetoday.com.au/vertigovideos) and the scientific evidence is convincing [Epley vs. sham maneuver, complete resolution of vertigo: OR 4.42 (95% CI 2.62–7.44); Epley vs. sham maneuver, conversion of Dix-Hallpike: OR 9.62 (95% CI 6.0–15.42)] (32).

Another effective, safe, and neglected tool is vestibular rehabilitation (VR; *tool #8*). VR is an exercise based treatment that gradually stimulates the vestibular system and vestibular compensation (34). Chronic vertigo occurs when natural vestibular compensation fails (35). Although a clear definition is lacking (36), chronic vertigo is—based on clinical course and expert opinion—often defined as symptoms persisting more than 1 month (17, 37). In primary care, physicians can refer patients to a specialized physiotherapist for VR. Despite the scientific evidence for VR, <10% of PCPs in the Netherlands and UK reported its use (33, 38). As this may be due to a lack of availability or access to VR (38), the University of Southampton developed a freely available online VR intervention (https://balance.lifeguidehealth.org). This online VR intervention was investigated among two different cohorts in primary care (*n* = 296 and *n* = 322, respectively), showing both reduction of dizziness and dizziness-related impairment after 3 and 6 months (39, 40). Being easily accessible, safe, effective and low cost, online VR has the potential to substantially improve the quality of life for a largely undertreated group of patients.

Although nowadays VR is the preferred treatment for chronic vertigo according to US, Dutch, and UK clinical practice guidelines (17, 41–43), anti-vertigo drugs like betahistine are still regularly prescribed. According to a large observational study, betahistine was initially prescribed to more than two thirds of vertigo patients in general practice and was still being used after 6 months (44). This enthusiastic prescribing contradicts with the state of the science, though, as a recent Cochrane review showed only weak evidence for the effectiveness of betahistine to treat chronic vertigo (45). Also, long term prophylactic treatment with betahistine does not change the time course of vertigo episodes related to Meniere's disease compared with placebo (46). When using GRADE methodology to compare VR (4 RCTs, *n* = 565 adults with different causes of chronic vertigo) with betahistine (11 RCTs, *n* = 606 adults with different causes of chronic vertigo), there is a difference in effectiveness and quality of evidence: vertigo patients receiving VR reported higher improvement compared to sham/no treatment [odds ratio 2.67 (95% CI 1.85–3.86); moderate quality of evidence], whereas vertigo patients treated with betahistine reported limited improvement compared to placebo [risk ratio 1.30 (95% CI 1.05–1.60); low quality of evidence]. In contrast to VR, none of the betahistine trials was conducted in primary care, which limits the generalizability (47). In short, when treating chronic vertigo, use exercise not drugs (*tool #9*).

## Prognosis

Until present, many risk factors of handicapping dizziness and/or vertigo have been identified, like chronic dizziness, daily dizziness, activity limitation or avoidance due to dizziness, anxiety or depression, polypharmacy, and impaired functional mobility (48–51). One of the most powerful predictors of an unfavorable course of dizziness, though, is significant impairment at baseline as measured with the Dizziness Handicap Inventory (DHI) (49, 52). The DHI is a 25-item self-report questionnaire, developed to measure impairment due to vestibular symptoms (53). Nowadays, it has been translated in at least 17 languages and considered to be the most used vestibular PROM (54). However, the length of the DHI limits its use in daily clinical practice. Therefore, the abbreviated 10-item DHI-S questionnaire (fill-in time ± 2 min) was developed in 1998 (55). During a psychometric evaluation in primary care, the DHI-S showed excellent criterion validity, test-retest reliability, and responsiveness (56). Recently, a prediction study with external validation in primary care showed that the ability of the DHI-S to identify patients at risk of an unfavorable course of dizziness improved when combined with the predictors age, history of arrhythmia, and looking up as a provoking factor (area under the curve after external validation = 0.78) (52). Given the fact that—in addition to its prognostic qualities—the DHI-S provides information on current handicap and can be used to monitor treatment effect, we believe that this questionnaire should not be missed in the vestibular toolkit of the PCP (*tool #10*).

## Conclusions

In this article, we present ten vestibular tools for primary care. PCPs can use these tools to improve diagnosis, treatment, and prognosis of vestibular symptoms. All tools are readily available and do not require intensive training. By simplifying proper management of vestibular symptoms, we hope that PCPs will embrace dizziness as an exciting symptom.

## Author Contributions

OM and VV wrote and approved the manuscript. All authors contributed to the article and approved the submitted version.

## Conflict of Interest

The authors declare that the research was conducted in the absence of any commercial or financial relationships that could be construed as a potential conflict of interest.
